# Housing Design and Community Care: How Home Modifications Reduce Care Needs of Older People and People with Disability

**DOI:** 10.3390/ijerph16111951

**Published:** 2019-06-01

**Authors:** Phillippa Carnemolla, Catherine Bridge

**Affiliations:** 1 School of Built Environment, Faculty of Design, Architecture and Building, University of Technology Sydney, Harris St, Ultimo, NSW 2007, Australia; 2 Faculty of Built Environment, University of New South Wales, Kensington, NSW 2052, Australia; c.bridge@unsw.edu.au

**Keywords:** housing, informal care, formal care, home modification, disability, ageing, accessibility

## Abstract

The extent to which housing design can minimise levels of community caregiving has remained largely unmeasured. This paper reports the potential for home modifications to reduce caregiving in the peoples’ homes, particularly older people and people with a disability. It contributes to new knowledge in understanding how housing can play a role in community caregiving and acknowledges the role of the built environment in managing care levels in ageing societies. This paper analyses self-reported care data from 157 Australian community care recipients (average age: 72 years) who had received home modifications within the past 6 months. A before/after comparison of care provided revealed that home modifications reduced hours of care provided by 42% per week. More detailed analysis revealed that the positive association of home modifications with care reduction is stronger with informal care (46% reduction) followed by formal care (16% reduction). These results suggest the role that home modifications, and housing design in general, play in reducing care needs in a community setting.

## 1. Introduction

The design and construction of housing impacts the quality of life and independence of a population, particularly one that is ageing [1]. The layout and structure of a home can trigger the need for paid care in the home or having to leave home to enter an aged care facility. Therefore, understanding the relationships between housing and health will help to improve the sustainability of community care models that support the independence and wellbeing of older people living at home.

The research presented in this paper examines changes in care requirements following home modifications for people ageing at home and receiving community care. “Home modification” describes “structural changes made to the homes of older people and people living with a disability” [2]. Home modifications are typically prescribed by an occupational therapist and are designed to support a person’s ability to live independently at home.

The potential for change to the built environment to reduce levels of disability, reduce healthcare cost and improve quality of life has been well documented [3,4]. However, the way in which change to the built environment relates directly to the need for care in the home is underexplored and unmeasured. Where related research has been conducted, it is difficult to synthesise due to the heterogenous nature of the research designs and the variations in what and how an intervention is measured. A systematic review of home modification evidence found that most related studies are multifactorial, where home modifications form a small part of an integrated care or health service approach. These multifactorial studies have made the effects of changes to buildings themselves difficult to predict and impossible to separate from results [2].

As we experience increasing need for care within our communities, a result of the effect of demographic changes and de-institutionalisation [5,6,7], there is a need to understand what role housing can play in reducing levels of care. This paper seeks to fill this gap by presenting research that measures changes in caregiving resulting directly from home modifications involving structural improvements to accessibility and safety. By comparing weekly care hours reported in the homes of a sample of 157 community care clients both before and after their home modifications, this paper provides an analysis of both formal (paid) and informal (unpaid) care in the context of improved housing conditions.

The paper begins by defining what is meant by the term “home modification” and highlighting alternative terminology. This is followed by an overview of the diverse evidence base on the effect of home modification. It then goes on to describe the study design, which involves the measurement of changes in weekly care hours provided in the home following home modifications. The results are then presented in the form of the demographic descriptive statistics followed by the care data, which indicates that home modifications directly resulted in community care saving, both informal and formal. Finally, the conclusion section summarises the findings on home modifications and care in the context of the sample.

### 1.1. Home Modifications

Home modifications are defined as “changes made to the home environment to help people to be more independent and safe in their own home and reduce any risk of injury to their carers and careworkers” [8]. Home modifications present a unique opportunity to directly measure how housing practices impact health and care as they are increasingly recognised as an effective policy and practice response to support frail, older people and those living with a disability to remain at home [9,10]. Exploring how home modification affects care outcomes acknowledges the role of housing in managing problems associated with maintaining levels of care in an ageing society where community care is promoted and preferred. 

Research into home modifications is conducted across the fields of housing and health. A number of studies have described the dynamic of the interdisciplinary nature of the topic “complex” [11,12]. Within the evidence base there are a range of terms being used to describe home modifications, including housing adaptation, assistive technology, environmental intervention and home adaptation. There are also some inconsistencies in the definition and scope of the interventions, with some studies including personal technology or the moving of furniture as part of the intervention.

Home modifications are themselves diverse in design and purpose, and can include major structural changes such as adding ramps, lifts or widening doors. They can also include minor, non-structural additions including assistive devices inside or outside the dwelling such as grab rails and handrails. 

Given the shift away from institutional care towards a reliance on home-based or community care internationally [13], research that considers and measures the benefits, both social and economic, of the built environment in the context of care provision will help to guide policies that will improve outcomes and the efficiencies of a community care model. This evidence on home modifications forms the foundation of an important economic argument for the ongoing provision of care in the community, namely how capital investment in housing might reduce the ongoing need for (and ongoing costs of) care. 

Scoping of the literature on home modifications reveals a variety of themes, including relationships with fall prevention, the ageing process, wellbeing, improved function or independence, physical health and wellbeing, caregiving and economic effectiveness. An overview of the evidence follows, and gives a picture of the multidimensional ways that the built environment impacts people’s ability to live independently in their own home, despite health changes.

#### 1.1.1. Preventing Falls and Improving Safety

A number of studies investigate the relationship between home modifications and fall prevention in some way. Most found positive evidence that a home modification intervention can reduce the likelihood of a fall or injury occurring [14,15,16,17,18]. However, a review by Wahl et al. [19] found no evidence of effect on fall prevention following home modification intervention.

#### 1.1.2. Improved Function and Independence

There is a collection of research that examines the relationship between home modifications and improved function and independence. A number of studies have found evidence of a relationship between home modifications and improved function (by reducing difficulty regarding activities of daily living) [19,20,21,22,23,24]. The research by Sheffield (2013) found there was no link between home modifications and function but did find evidence of a reduction in fear of falling and increases in safety [25]. Szanton et al. [23] also found improvements in the ability to provide self-care following home modifications. 

#### 1.1.3. Physical Health and Wellbeing

Two random controlled trials directly measured the effect of home modifications on wellbeing and determined evidence of a relationship between home modifications and increased quality of life [26,27]. Studies by Heywood and Turner [28] and Andrich, Ferrario & Moi [29] employ qualitative methods to demonstrate the existence of a relationship between home modifications and wellbeing.

#### 1.1.4. Ageing Process

Studies that investigate housing and the ageing process tend to focus on either supporting ageing in place in general or reducing the progression of frailty. Mitoku and Shimanouchi [30] reports on the evidence that home modifications slow the progression of frailty. Three studies report on the effect of home modifications on ageing in place [31,32,33]. Tanner et al. [34] reports that modifications contribute to the meaning of home for older people. Renaut, Ogg, Petite and Chamahian [35] found more neutral results and found that home modifications do not necessarily contribute to how people adapt to home. The study by Ahn and Hegde [36] did not find a link between home modifications and home environment satisfaction.

#### 1.1.5. Caregiving

In most of the studies that have investigated caregiving and housing changes, home modifications are included as part of a larger study of interventions, e.g., assistive technology. These studies found a positive association and reported a reduced need for care [21,22,37], support of caregiving practices [38,39] or offsetting of institutionalisation [40].

Despite this extensive body of evidence, one of the main gaps to overcome is the lack of research that isolates home modifications as a single intervention. The prevalence of multifactorial interventions in the studies means that the effect of housing on these outcomes cannot be isolated. The project research design presented in this paper has addressed this issue.

### 1.2. Concepts of Community Care and Housing

In managing the needs of older people or people living with a disability, the developed world continues to move away from institutionalised models of caregiving and towards individualised care solutions [41]. This has meant a move away from largescale, institutional built environments specifically dedicated to high-volume caregiving, towards all levels and types of housing and accommodation having the propensity to accommodate levels of care provision. Caregiving in the home has become one of the ways to bridge the human performance gaps created when a person’s home environment is not adequate for their level of functioning. Understanding the interactions between housing and caregiving reveals why buildings play a role in supporting care provision, and accessibility (or lack thereof) becomes a critical trigger for people being forced into assisted living and residential care settings.

One of the reasons older people and people with a disability are required to transition from living at home to an assisted living facility or residential care setting lies in feelings of incompetence to complete tasks independently (increased caregiving) and unsuitable housing design [42]. Countries like Australia, and others throughout the developed world, continue to experience increasing demand for community care services and the relationship between care and housing is poorly understood. Set amongst a housing stock of predominantly inaccessible, older buildings, the general lack of suitable housing for people with care needs further highlights how, for people living with a disability at any age, the issues of finding accessible and secure housing to complement specific care needs long-term are an ongoing concern [43].

In order to understand how home modifications substitute for caregiving, it is important to classify what is meant by community care and the types of community care that can be provided in the home. Community care encompasses a wide range of tasks, intentions and health and welfare frameworks. The World Health Organisation defines community care as the “services and support to help people with care needs to live as independently as possible in their communities” [44]. In the Western context, community care also refers to the set of legal and practical arrangements implemented by health and social welfare agencies to facilitate the relative independence of a person within community setting.

Community care is provided by either paid care staff (formal care) or by family members (informal care). Types of community care can include personal care (such as toileting, washing or feeding), medical care, or domestic assistance (housework).

### 1.3. Substitution of Care

Along with the understanding that care formats can blend with each other is the theory that in some cases, care types will substitute for each other [45,46]. This understanding forms the premise of the present study design that tests whether building construction in the form of home modifications can provide an alternative solution to community care (formal and informal care) by supporting self-care and maintaining independence levels in the home.

There is a body of evidence that elucidates the effects of home modifications to improve independence health and wellbeing; however, there appears to be far less empirical research into whether there is any substitution of care occurring following modifications to housing. What is clear in the evidence, however, is an understanding of the amount of care being provided within a community setting, predominantly in people’s homes. In an Australian context, where the research reported in this paper was undertaken, the Australian Bureau of Statistics (ABS) Caring in the Community data indicates that 40% of all Australian households include a person who is providing informal care [47]. In earlier ABS reports, 94% of older people living in the community who required in-home care received assistance from the informal care network [48]. Approximately 33% of older people receiving support received a combination of informal carers and formal services, implying that both care types can and do function interdependently and blend with each other [49]. This leaves a very small percentage relying solely on formal care assistance provided by a community organisation or a health professional.

An over reliance on informal care can lead to increased stress and decreased health for carers [50] and can have implications on human capital by taking people out of the workforce [51,52,53]. One of the major impacts of informal caring is a lower probability of employment on the part of the carer [54,55]. Formal care is also under increased pressure due to changes in how care is provided within families, as well as the desire for older people requiring care to “age in place” at home. Increasing labour shortages in the care industry and the high demand for care (which currently exceeds the supply) are identified as crucial areas of aged care policy requiring alternative and innovative care solutions [56,57]. Understanding of the impact of high levels of care need in the community leads to a realisation of the significance of this study. For example, what if capital investment in the built environment could substitute for ongoing care demand?

## 2. Materials and Methods

This paper focuses on the measurement of care changes before and after home modifications. The study design is comprised of a single-arm and incorporates a cross-sectional data capture of participants “pre and post” survey responses. The study surveyed a sample of 157 people who had received home modifications via an Australian government-subsidised home modification program. In order to be eligible to be included in the study, participants had to be community-dwelling clients of a government-subsidised community care program known as Home and Community Care (HACC) who had received home modifications within the previous 6 months.

Having participants sourced from within a government-subsidised program of home modification means that privately-funded home modifications are excluded from this research. There are two reasons for this approach. First, there was no central registry of privately-funded home modifications from which to source participants effectively. Second, targeting government-registered home modifications enabled access to additional data of accurate and consistent home modification costs and type, as well as the health information for each participant.

A survey was designed to examine the home modification experiences of participants and sought care hours before and after home modification, self-reported as weekly hours of informal and formal care. The surveys were distributed via home modification providers in NSW, Australia. Surveys were posted as part of a standard follow-up to recent recipients of home modifications where all modifications had been completed within the previous six months.

The survey included four multiple-choice questions requesting demographic information such as tenure and income status. This was followed by a section on formal and informal care associated with bathing, toileting or moving around the house. Care was separated into categories of informal and formal, and recorded as self-reported hours per week. The final question was open-ended, seeking people’s comments on how home modifications may have changed their health and/or care needs.

The research and survey instrument was introduced to prospective participants by the prescribing occupational therapists at the time of the home modification completion. The surveys were separately posted directly to prospective participants along with an information sheet explaining key care terms “formal” and “informal” as “paid” and “unpaid” care, respectively. These instructions included explanation of how to fill in the survey correctly and self-report hours of care on the form.

Participants recalled their pre-home modification care as well as their current (post-home modification) care. Although this introduced the possibility of recall bias, it was considered the most effective way of conducting an exploratory study of changing care needs and minimising the issues of participant attrition, which is a heightened concern when interacting with frail, older people. The self-reported data was also potentially influenced by social compliance bias (also known as social desirability bias). The potential for responses to be more favourable to please or thank the home modification provider was recognised and this bias was minimised in two ways: first, by assurances of anonymity, and second the survey was returned directly to the researcher, not the home modification provider.

Parameters around the housing aspect of the study were important. The definition of home modification was made clear from the outset as being structural changes to a person’s home to enable them to remain living in the community. The HACC program from which the participants were sourced provided interventions within the agreed definition of home modification. The survey responses were matched to deidentified client files that included information on the type of home modifications installed. This meant that home modifications included in the study were analysed by type. 

### Sample Response Rate

One hundred and sixty-five home modification recipients responded to the survey. After eliminating eight incomplete or invalid responses, a total of 157 respondents were included in the analysis. This yielded a survey response rate of 24.1% (157 participants out of a sample of 650 eligible participants). 

Ethics approval was granted by the University of New South Wales Human Research Ethics Committee (UNSW HREC) in November 2014 (ETH-125082).

## 3. Results

In this section, the survey results are documented. First, descriptive statistics of the sample are presented. These include an analysis of gender, age, housing tenure, living arrangement and source of income. Second, the care hour data is presented in terms of care type (informal and formal care) for before and after home modifications. 

### 3.1. Descriptive Statistics

The descriptive statistics for the sample are set out in Table 1.

The average age of the sample was found to be 72 years at the time of data collection. In an analysis of gender within the sample, females outnumbered males by 5:4, with 85 participants in the study being female (54.1%). The survey requested information on housing tenure and Table 1 shows that the sample were likely to be owner/occupiers of their own homes (94.9%).

Reporting on the living arrangements of participants, the majority lived with a spouse or partner (53.5%); while 19.1% were living with family or friends and 26.8% of respondents reported living alone. This is a lower representation than reported in the HACC Minimum Data Set, where 42% of the NSW HACC client population were reported as living alone.

Respondents were asked about their main source of income. A majority of the participants (65.0%) were supported by a full aged pension; 17.83% received a disability support pension and 10.19% received a part aged pension. A smaller percentage (1.27%) was supported by a carer’s allowance. A total of 5.8% were either self-funded retires (5.1%) or on a full-time wage or salary (0.64%). At the time of the data collection, the study sample were overwhelmingly (94.3%) dependent on government welfare as their main source of income, signalling economic vulnerability. 

### 3.2. Home Modification Results

**Figure 1:**
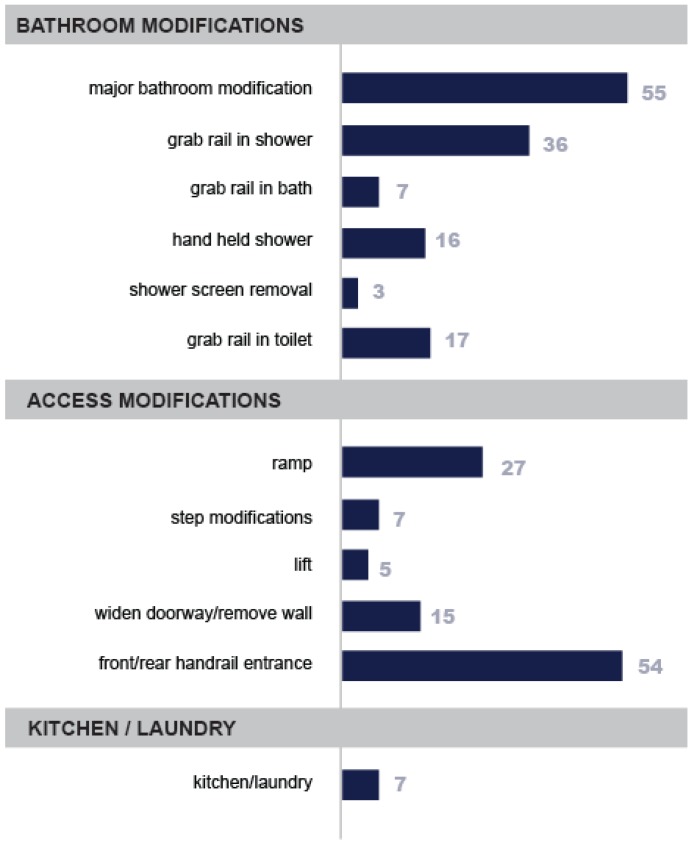
Home modification frequency by type. Note: Where participants received major bathroom modifications, they were not included in the count for other, itemised bathroom modifications. Kitchen and laundry modifications refer to cabinet height/design changes, widening of work areas or mounting of appliances for easier access.

Data was captured about the type and location of the home modifications within the sample, these are illustrated in Figure 1 below. This enabled a picture to be drawn of the range of modifications that were included under the banner of “home modifications” and where in the house they were located. Major bathroom modifications were the most prevalent single type, followed by handrails for the front or rear access to the house.

### 3.3. Care Results

The care data results revealed whether a home modification changed the amount of care provided in the home. The before and after care was measured as weekly hours and tested as pre–post paired samples for significance, using SPSS software (IBM Corp. Released 2013. IBM SPSS Statistics for Windows, Version 22.0. Armonk, NY, USA). The results of the paired *t*-test indicated that for all care types, changes in care hours following home modifications were statistically significant. Even for the least significant result (formal care), *p* was 0.04, which is <0.05.

For informal care provision, there is strong evidence (*t* = 6.39, *p* = 0.00) that home modifications reduced the need for informal care. In this data set, it reduced informal care hours by approximately 6 h per week, with a 95% confidence interval of between 4.12 and 7.8 h per week savings.

For formal care provision, there is evidence (t = 2.08, *p* = 0.04) that home modifications reduced the need. In this data set, it reduced formal care hours by approximately 0.36 h per week, with a 95% confidence interval of between 0.02 and 0.7 h per week savings.

For total care provision (formal + informal care) there is strong evidence (t = 6.8, *p* = 0.00) that home modifications reduced the need for care. In this data set, it reduced total care hours by approximately 6.32 h per week, with a 95% confidence interval of between 4.48 and 8.15 h per week savings.

**Figure 2:**
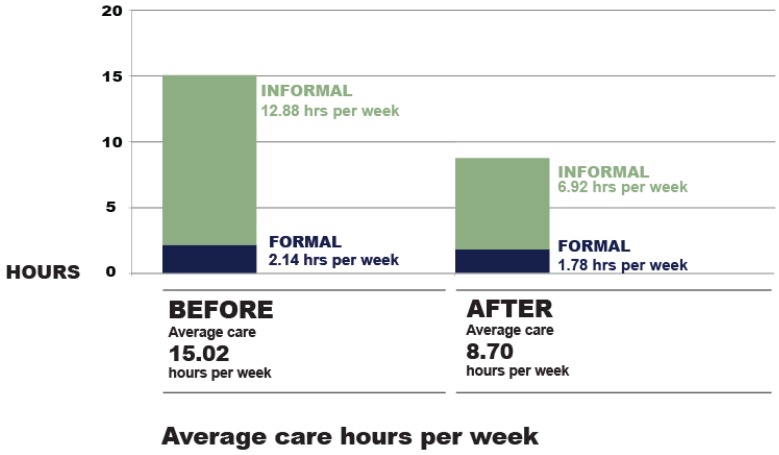
Analysis of care hours before and after home modifications.

Figure 2 shows the pre–post care results as a comparison of combined formal and informal care before home modifications (total 15.02 h per week) and combined formal and informal care after home modifications (total 8.7 h per week):

## 4. Discussion

Changes in care provided in the home tell a story about overall independence, autonomy and the ability to maintain current housing situations. Savings in informal care following a home modification reveal a story about both carer and recipient, directly reflecting factors such as human capital and carer stress in a community. Alternatively, changes to formal care reveal a story about costs to government and health systems.

The research findings suggest that home modifications support a model of self-care and substitute for both informal and formal care provided in the home. This relationship is stronger in informal care than in formal care. The reason behind the higher sensitivity of informal care hours over formal care hours following home modifications is unclear; however, there are a number of possible explanations. First, informal care is provided in such a way that it can respond more flexibly to changes in need in the home. In contrast, government-subsidised formal care has assessment, administration and eligibility requirements that are managed separately from the home modification interventions, and therefore are less likely to be flexible. Second, it is possible that formal care variations may not be fully captured by the survey because of the time lag between receiving home modifications and administering changes to formal care. Third, given the shortage of care services and difficulty in obtaining government-subsidised care, people may be reluctant to give up any care they currently receive, preferring to use it in other ways. This in turn implies a level of unmet need for care in the community, which has been acknowledged in previous studies [58].

The study sample, while also participating in a government-subsidised program of home modifications, also proved to be overwhelmingly dependent on government welfare, signalling economic vulnerability. At the time of the data collection, eligibility into the home modifications program was not dependent on any means testing; however, it was prioritised according to vulnerability to residential care. 

The study sample were also found to be overwhelmingly the owners/occupiers of their own homes (94%). It signals that older, private renters are less likely to access home modification services compared to homeowners. Although home ownership has been the most common tenure in older populations in Australia, this is starting to change [59], and suggests the need for ongoing investigation into home modification rights and access to services. 

Home modifications have been studied across a number of fields (including housing and health) and their effects are diverse. As an intervention, they have been found to positively impact the independence, autonomy, self-care and wellbeing of people living at home with care (older people as well as people living with disability) [2]. This study further supports the evidence base of the home modification literature and provides the previously unmeasured effect of home modifications on direct hours of care provided within the home. 

The findings in this exploratory study represent a significant contribution to understanding the built environment as it relates to human performance and human impact for two reasons. First, they highlight how important buildings are to the ongoing independence of the populations. Second, they raise the possibility that a capital investment in the built environment can offer ongoing returns in care cost savings. This economic argument is in line with other studies [18,60].

Having acknowledged some of the limitations previously, the present study design is exploratory. It makes a significant contribution to the built environment research and housing research because the care data is collected as primary data, specifically for this study as it relates to built environment change. This means that home modifications are measured as a single factor housing intervention, without other interventions, such as therapies or assistive technology. The exclusion of other interventions means the implications of housing change in the results are transparent.

## 5. Conclusions

The findings in this study confirm the positive human impact effects of modifying housing, in particular bathrooms, providing evidence that home modifications directly support those needing care and reduce amount of care required in the home. One of this study’s strengths is that it uses primary data, drawing on original, self-reported care hour data for both before and after the home modifications, enabling a previously under-explored interaction between home modification and community care.

The data analysed in this paper synthesises knowledge in the fields of health and built environment, acknowledging the built environment as potentially playing a central role in problems associated with maintaining care and independence in a home setting. In terms of the effectiveness of home modifications, the study demonstrates that installing home modifications directly results in a reduction in the need for care in the home by up to 46%. This is a significant result, comprised predominantly of savings made from the reduction in informal care hours. 

The results confirm evidence of a relationship across housing and care, and therefore support the case for housing policy and health care reforms to be considered concurrently. Other expected benefits of reducing the need for both formal and informal care in the home include a reduced cost of caregiving, and the ability for informal caregivers to work outside the home impacting available human capital in the workforce. These benefits warrant further research in the context of built environment effects.

The research has important implications for design and construction approaches and has relevance for built environment professionals, supporting an understanding of the broader “human impact” implications of populations choosing to stay at home as they continue to age. Further research is warranted to consolidate the interdisciplinary metrics across built environment and health care. The economic benefits of a capital investment in housing is the subject of a cost utility analysis of home modifications currently being planned for publication.

## Figures and Tables

**Table 1:** Descriptive Statistics.

	Count	Percentage of Total Sample
*Mean age (years)*	72	
*Gender*	157	
Female	85	54.1%
Male	72	45.9%
*Housing tenure*		
Being purchased	2	1.27%
Fully owned	149	94.90%
Live with family members	2	1.27%
Own caravan/annex and rent site	1	0.64%
Rental (private)	2	1.27%
Retirement village	1	0.64%
*Living arrangements*		
Lives alone	43	26.75%
Lives with a spouse or partner	84	53.50%
Lives with family or friends	30	19.11%
*Income source*		
Carers allowance	2	1.27%
Disability support pension	28	17.83%
Full aged pension	102	64.97%
Part aged pension	16	10.19%
Self-funded retiree	8	5.10%
Wage or salary (full time)	1	0.64%

## References

[1] Oswald F., Wahl H.W., Schilling O., Nygren C., Fänge A., Sixsmith A., Iwarsson S. (2007). Relationships between housing and healthy aging in very old age. Gerontologist.

[2] Carnemolla P., Bridge C. (2018). A scoping review of home modifications interventions—Mapping the evidence base. Indoor Built Environ..

[3] Altman B., Barnartt S. (2014). Environmental Contexts and Disability.

[4] Straton J., Saunders N., Broe T., Brown W., Earle L., Gregory B. Promoting Healthy Ageing in Australia. http://www.dest.gov.au/science/pmseic/documents/promotinghealthyageingreport.doc.

[5] Cangiano A., Shutes I., Spencer S., Leeson G. (2009). Migrant Care Workers in Ageing Societies: Research Findings in the UK.

[6] Kelly J. (2013). Industry View ACSA: Calling Attention to Ageing.

[7] Simonazzi A. (2009). Care regimes and national employment models. Camb. J. Econ..

[8] Adams T., Bridge C., Carnemolla P., McNamara N., Quinn J. (2014). Consumer Factsheet: Arranging Home Modifications. Consumer Factsheet Series.

[9] Fänge A., Iwarsson S. (2005). Changes in ADL dependence and aspects of usability following housing adaptation—A longitudinal perspective. Am. J. Occupational Ther..

[10] Pynoos J. (1993). Toward a national policy on home modification. Technol. Disabil..

[11] Johansson K., Lilja M., Petersson I., Borell L. (2007). Performance of activities of daily living in a sample of applicants for home modification services. Scand. J. Occup. Ther..

[12] Lord S., Menz H., Sherrington C. (2006). Home environment risk factors for falls in older people and the efficacy of home modifications. Age Ageing.

[13] Coyte P.C., McKeever P. (2001). Home care in Canada: Passing the buck. Can. J. Nursing Res.

[14] Chang J.T., Morton S.C., Rubenstein L.Z., Mojica W.A., Maglione M., Suttorp M.J., Shekelle P.G. (2004). Interventions for the prevention of falls in older adults: Systematic review and meta-analysis of randomised clinical trials. BMJ.

[15] Clemson L., Mackenzie L., Ballinger C., Close J., Cumming R. (2008). Environmental interventions to prevent falls in community-dwelling older people a meta-analysis of randomized trials. J. Aging Health.

[16] Tse T. (2005). The environment and falls prevention: Do environmental modifications make a difference?. Aust. Occup. Ther. J..

[17] Turner S., Arthur G., Lyons R.A., Weightman A.L., Mann M.K., Jones S.J., Lannon S., Rolfe B., Kemp A., Johansen A. (2011). Modification of the home environment for the reduction of injuries. Cochrane Database Syst. Rev..

[18] Powell J., Mackintosh S., Bird E., Ige J., Garrett H., Roys M. The role of home adaptations in later life. Centre for Ageing Better, University of the West of England, BRE. https://www.ageing-better.org.uk/sites/default/files/2017-12/The%20role%20of%20home%20adaptations%20in%20improving%20later%20life.pdf.

[19] Wahl H., Fänge A., Oswald F., Gitlin L., Iwarsson S. (2009). The home environment and disability-related outcomes in aging individuals: What is the empirical evidence?. Gerontologist.

[20] Chase C., Mann K., Wasek S., Arbesman M. (2012). Systematic review of the effect of home modification and fall prevention programs on falls and the performance of community-dwelling older adults. Am. J. Occup. Ther..

[21] Gitlin L., Corcoran M., Winter L., Boyce A., Hauck W. (2001). A randomized, controlled trial of a home environmental intervention: Effect on efficacy and upset in caregivers and on daily function of persons with dementia. Gerontologist.

[22] Mann W., Ottenbacher K., Fraas L., Tomita M., Granger C. (1999). Effectiveness of assistive technology and environmental interventions in maintaining independence and reducing home care costs for the frail elderly: A randomized controlled trial. Arch. Family Med..

[23] Szanton S., Wolff J., Leff B., Thorpe R., Tanner E., Boyd C., Gitlin L. (2014). CAPABLE trial: A randomized controlled trial of nurse, occupational therapist and handyman to reduce disability among older adults: Rationale and design. Contemp. Clin. Trials.

[24] Wilson D., Mitchell J., Kemp B., Adkins R., Mann W. (2009). Effects of assistive technology on functional decline in people aging with a disability. Assist. Technol..

[25] Sheffield C., Smith C., Becker M. (2013). Evaluation of an Agency-Based Occupational Therapy Intervention to Facilitate Aging in Place. Gerontologist.

[26] Ahmad J., Shakil-ur-Rehman S., Sibtain F. (2013). Effectiveness of home modification on quality of life on wheel chair user paraplegic population. Rawal Med. J..

[27] Lin M., Wolf S., Hwang H., Gong S., Chen C. (2007). A randomized, controlled trial of fall prevention programs and quality of life in older fallers. J. Am. Geriatr. Soc..

[28] Heywood F., Turner L. (2007). Better Outcomes, Lower Costs. Implications for Health and Social Care Budgets of Investment in Housing Adaptations, Improvements and Equipment: Review of the Evidence.

[29] Andrich R., Ferrario M., Moi M. (1998). A model of cost-outcome analysis for assistive technology. Disability Rehabil..

[30] Mitoku K., Shimanouchi S. (2014). Home Modification and Prevention of Frailty Progression in Older Adults: A Japanese Prospective Cohort Study. J. Gerantol. Nursing.

[31] Hwang E., Cummings L., Sixsmith A., Sixsmith J. (2011). Impacts of home modifications on aging-in-place. J. Housing Elderly.

[32] Safran-Norton C. (2010). Physical home environment as a determinant of aging in place for different types of elderly households. J. Housing Elderly.

[33] Pettersson C., Löfqvist C., Malmgren Fänge A. (2012). Clients’ experiences of housing adaptations: A longitudinal mixed-methods study. Disability Rehabil..

[34] Tanner B., Tilse C., De Jonge D. (2008). Restoring and sustaining home: The impact of home modifications on the meaning of home for older people. J. Housing Elderly.

[35] Renaut S., Ogg J., Petite S., Chamahian A. (2014). Home environments and adaptations in the context of ageing. Ageing Soc..

[36] Ahn M., Hegde A. (2011). Perceived aspects of home environment and home modifications by older people living in rural areas. J. Housing Elderly.

[37] Agree E., Freedman V., Cornman J., Wolf D., Marcott J. (2005). Reconsidering substitution in long-term care: When does assistive technology take the place of personal care?. J. Gerontol. Series B Psychol. Sci. Soc. Sci..

[38] Anderson W., Wiener J. (2013). The impact of assistive technologies on formal and informal home care. Gerontologist.

[39] Naik A.D., Gill T. (2005). Underutilization of environmental adaptations for bathing in community-living older persons. J. Am. Geriatrics Soc..

[40] Newman S., Struyk R., Wright P., Rice M. (1990). Overwhelming odds: Caregiving and the risk of institutionalization. J. Gerontol..

[41] Hemingway L. (2011). Disabled People and Housing: Choices, Opportunities and Barriers.

[42] Golant S.M. (2011). The quest for residential normalcy by older adults: Relocation but one pathway. J. Aging Stud..

[43] Beer A., Faulkner D. (2008). The Housing Careers of People with a Disability and Carers of People with a Disability.

[44] Andrews G., Faulkner D., Andrews M. (2004). A Glossary of Terms for Community Health Care and Services for Older Persons.

[45] Agree E., Freedman V. (2000). Incorporating assistive devices into community-based long-term care: An analysis of the potential for substitution and supplementation. J. Aging Health.

[46] Bonsang E. (2009). Does informal care from children to their elderly parents substitute for formal care in Europe?. J. Health Econ..

[47] Australian Bureau of Statistics (2012). Disability, Ageing and Carers, Australia: Summary of Findings, 2012.

[48] Australian Bureau of Statistics (1995). Focus on Families: Caring Infamilies—Support for Persons Who are Older or Have Disabilities.

[49] Liu K., Manton K., Aragon C. (2000). Changes in home care use by disabled elderly persons 1982–1994. J. Gerontol. Series B Psychol. Sci. Soc. Sci..

[50] Pinquart M., Sörensen S. (2007). Correlates of physical health of informal caregivers: A meta-analysis. J. Gerontol. Series B Psychol. Sci. Soc. Sci..

[51] Bittman M., Hill T., Thomson C. (2007). The impact of caring on informal carers’ employment, income and earnings: A longitudinal approach. Aust. J. Soc. Issues.

[52] Bolin K., Lindgren B., Lundborg P. (2008). Informal and formal care among single-living elderly in Europe. Health Econ..

[53] Van Houtven C.H., Coe N.B., Skira M.M. (2013). The effect of informal care on work and wages. J. Health Econ..

[54] Carers Australia (2014). Combining Work and Care: The Benefits to Carers and the Economy. Work and Care: The Necessary Investment.

[55] Davies R., Gray C. (2009). Care pathways and designing the health-care built environment: An explanatory framework. Int. J. Care Pathw..

[56] Ham C., Dixon A., Brooke B. (2012). Transforming the Delivery of Health and Social Care. The Case for Fundamental Change.

[57] Desai M., Lentzner H., Weeks J. (2001). Unmet need for personal assistance with activities of daily living among older adults. Gerontologist.

[58] Lansley P., McCreadie C., Tinker A. (2004). Can adapting the homes of older people and providing assistive technology pay its way?. Age Ageing.

[59] Australian Government (2015). Housing Decisions of Older Australians, Productivity Commission Research Paper.

[60] Jutkowitz E., Gitlin L., Pizzi L., Lee E., Dennis M. (2012). Cost Effectiveness of a Home Based Intervention That Helps Functionally Vulnerable Older Adults Age in Place at Home. J. Aging Res..

